# Estimating Biomass and Canopy Height With LiDAR for Field Crop Breeding

**DOI:** 10.3389/fpls.2019.01145

**Published:** 2019-09-26

**Authors:** James D. C. Walter, James Edwards, Glenn McDonald, Haydn Kuchel

**Affiliations:** ^1^School of Agriculture, Food and Wine, The University of Adelaide, Glen Osmond, SA, Australia; ^2^Australian Grain Technologies Pty Ltd, Roseworthy, SA, Australia

**Keywords:** wheat, phenomics, high throughput phenotyping, field phenotyping, plant breeding

## Abstract

Above-ground biomass (AGB) is a trait with much potential for exploitation within wheat breeding programs and is linked closely to canopy height (CH). However, collecting phenotypic data for AGB and CH within breeding programs is labor intensive, and in the case of AGB, destructive and prone to assessment error. As a result, measuring these traits is seldom a priority for breeders, especially at the early stages of a selection program. LiDAR has been demonstrated as a sensor capable of collecting three-dimensional data from wheat field trials, and potentially suitable for providing objective, non-destructive, high-throughput estimates of AGB and CH for use by wheat breeders. The current study investigates the deployment of a LiDAR system on a ground-based high-throughput phenotyping platform in eight wheat field trials across southern Australia, for the non-destructive estimate of AGB and CH. LiDAR-derived measurements were compared to manual measurements of AGB and CH collected at each site and assessed for their suitability of application within a breeding program. Correlations between AGB and LiDAR Projected Volume (LPV) were generally strong (up to r = 0.86), as were correlations between CH and LiDAR Canopy Height (LCH) (up to r = 0.94). Heritability (H^2^) of LPV (H^2^ = 0.32–0.90) was observed to be greater than, or similar to, the heritability of AGB (H^2^ = 0.12–0.78) for the majority of measurements. A similar level of heritability was observed for LCH (H^2^ = 0.41–0.98) and CH (H^2^ = 0.49–0.98). Further to this, measurements of LPV and LCH were shown to be highly repeatable when collected from either the same or opposite direction of travel. LiDAR scans were collected at a rate of 2,400 plots per hour, with the potential to further increase throughput to 7,400 plots per hour. This research demonstrates the capability of LiDAR sensors to collect high-quality, non-destructive, repeatable measurements of AGB and CH suitable for use within both breeding and research programs.

## Introduction

In recent years there has been much discussion regarding the role of high-throughput phenotyping (HTP) technologies within field crop breeding programs, focused primarily on the potential of these technologies to reduce the current disparity between the amount of phenotype and genotype data available to breeders (Cobb et al., 2013; Araus and Cairns, 2014). There are three key aspects of these technologies which interest field crop breeders: i) the ability to collect data faster than traditional methods; ii) the ability to collect higher-quality objective data than traditional methods; and (iii) the ability to collect data which cannot be collected through existing methods. With these three aspects in mind, the trait of above-ground biomass (AGB) is a prime candidate to benefit from the potential advantages offered by HTP technologies.

Above-ground biomass is traditionally measured through laborious and destructive methods, requiring crop cuts to be collected from field plots and dried in an oven before being weighed to assess the dry biomass of each sample. This multi-step process is prone to error, from variability in the area within the plot sampled, to the potential loss of material while cutting, transporting, and handling samples. Furthermore, the destructive nature of crop cuts is undesirable within field crop breeding programs due to the loss of plot area and edge effects that influence plot yield. Despite the inconvenience of phenotyping AGB, it is an important trait of interest in many field crop breeding programs. For bread wheat (*Triticum aestivum* L.), AGB has been identified as a trait with much potential to exploit within breeding programs, particularly in relation to yield improvements through harvest index and radiation use efficiency (Reynolds et al., 2012), water use efficiency (Richards et al., 2002), drought tolerance (Fischer and Wood, 1979), as well as potential advantages in crop competitiveness (Zerner et al., 2016).

Of the sensors investigated to estimate AGB with HTP to date, one of the most promising is LiDAR, a laser-based sensor, from which raw data can be transformed into a three-dimensional (3D) point cloud. As AGB is a 3D trait in nature, point cloud data provides a logical advantage compared to two-dimensional sensors such as digital or multispectral cameras, to accurately account for and estimate AGB of field crops. Although there are other methods and technologies that can be used to generate point cloud data, such as digital images and photogrammetry techniques (Walter et al., 2018), LiDAR-based systems offer not only a high-throughput and high-density method of collecting such data, but also the possibility of penetrating and collecting measurements from within the crop canopy.

To date, few studies have investigated the use of LiDAR, or similar technologies, to estimate the AGB of field crops. Those that have, often used LiDAR-derived canopy height (CH) as a proxy of AGB (Long and McCallum, 2013; Pittman et al., 2015; Eitel et al., 2016). This approach may be suitable for large-scale biomass estimation, such as in commercial crops, but in cereal breeding programs there is often little variation in CH among breeding lines. Investigations into processing methods which utilize the 3D nature of LiDAR-derived point cloud data have been undertaken, with volume measurements of point clouds (Jimenez-Berni et al., 2018; Sun et al., 2018; Walter et al., 2018), and 3D indices (Jimenez-Berni et al., 2018) shown to correlate strongly to manually measured AGB. While the findings of these studies are promising, they have not fully investigated how these methods and data may be applied to field crop breeding programs. One particular shortcoming of these previous studies is that they were limited to a single environment and a relatively small number of plots, in contrast to commercial breeding programs, which operate across many environments and require many plots to be evaluated.

The current study investigates the deployment of a LiDAR-based system for the non-destructive estimation of AGB and CH across multiple environments, with this system based on the High-throughput Imaging Boom (HIB) described by Walter et al. (2019). The logistics of integrating such a system within a breeding program are discussed, along with the relevance of the data to breeding programs, particularly focusing on trait heritability and genetic and residual correlations. Though the current study takes place within a wheat breeding program, we believe this discussion is relevant to a wide variety of field crop breeding and research programs.

## Methods

### Site and Trial Design

To investigate the application of LiDAR sensors within a wheat breeding program, field trials were run across eight sites in southern Australia, encompassing a range of environments with differences in yield potential. The trial sites selected are used for the evaluation of germplasm by a commercial wheat breeding program and are representative of the environmental range over which wheat is grown in the region. Location and details of the eight sites are shown in **Table 1**.

**Table 1:** Location and details of the eight field trial sites present in the current study. Latitude and longitude are presented in the WGS84 datum.

Site		Latitude (°)	Longitude (°)	Mean crop above-ground biomass at ZGS 65 (t/ha)	Mean crop canopy height at ZGS 65 (cm)
Angas Valley	(AV)	−34.758645	139.241074	6.7	76.5
Booleroo	(BL)	−32.801685	138.296129	5.5	63.5
Kaniva	(KV)	−36.436664	141.197603	14.6	87.7
Minnipa	(MN)	−32.841374	135.156289	1.4	39.6
Pinnaroo	(PN)	−35.350264	141.066939	7.9	81.4
Roseworthy	(RS)	−34.526346	138.665595	11.1	92.8
Rudall	(RD)	−33.656549	136.141373	5.0	49.4
Winulta	(WT)	−34.253630	137.884995	12.4	88.2

Each trial consisted of eight bread wheat (*Triticum aestivum* L.) cultivars, grown in small plots and designed as a completely randomized factorial design, with factors of genotype and sample time and three replicates (192 plots total). More sampling times were allocated during experimental design than were ultimately utilized in the current study. Trials were uniquely randomized at each site and were specifically designed to provide large amounts of phenotypic variation for plant height and above-ground biomass. Cultivars selected for this purpose were: Axe, Beckom, Halberd, Krichauff, Scepter, Shield, Wyalkatchem, and Yitpi. Trials were located within large-scale wheat breeding sites (approximately 6 ha and 8,000 plots per site) and managed by Australian Grain Technologies (AGT). Plots were 3.2 × 1.32 m and consisted of five rows spaced at 25 cm.

### Manual Measurements

Manual measurements were collected across all sites during the growing season. Sample times differed between sites (**Table 2**), though all sites were sampled at anthesis — Zadoks growth scale 65 [ZGS 65 (Zadoks et al., 1974)] — as a measurement of maximum leaf biomass. Developmental rate differs between the varieties used in the current study, with flowering time spread approximately across a two week window. As such ZGS values assigned are nominal, and sample dates were determined when 50% of varieties were at, or had surpassed, the designated ZGS. This does not impact on the processing or analysis methods used for the purpose of comparing manual and digital measurements in the current study. At each sample time CH was measured in each plot, while AGB was measured in plots of the corresponding sample time. Canopy height was measured with a ruler at four randomly-selected points within each plot, with an average of these heights recorded to provide a representative CH. Above-ground biomass was collected from individual plots as two linear meters of plant material (1m from two adjacent rows) cut at ground level. Cuts were taken from the inner seed rows to avoid edge effects. Cuts from each plot were bundled, dried at 45ºC for two weeks, then weighed to obtain AGB. Due to the small size of plots sown in the trial it was impractical to take multiple AGB samples from individual plots. To circumvent this, sampling time was allocated as a factor within the trial design, such that each sampling time was undertaken within unique plots.

**Table 2:** Sample times and associated Zadoks growth scale for each of the sites in the current study.

Site	Sample time				
	*ZGS 31*	*ZGS 49*	*ZGS 59*	*ZGS 65*	*ZGS 96*
AV		✓		✓	
BL				✓	
KV				✓	
MN				✓	
PN				✓	
RS	✓	✓	✓	✓	✓
RD				✓	
WT	✓	✓		✓	

### LiDAR Measurements

**Figure 1:**
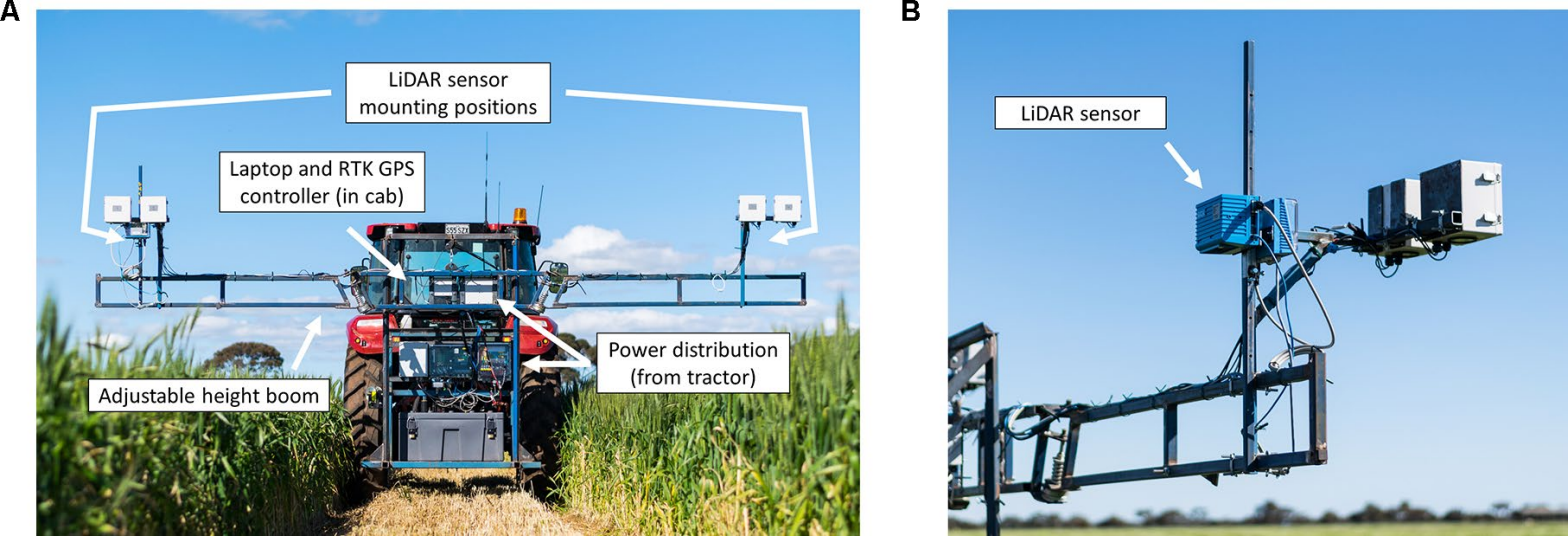
The tractor mounted LiDAR system used in the current study showing the boom system and LiDAR sensor mounting positions, with major components annotated **(A)** and a closer view of one of the mounted LiDAR sensors **(B)**.

The LiDAR sensors used in the current study were SICK LMS400-2000 (SICK AG, Waldkirch, Germany). These 2D sensors have a 70° field of view and are capable of scanning between 270–500 Hz at an angular resolution of 0.1°–1.0°, with a ± 4 mm systematic measurement error and a 3–10 mm statistical error, depending on remission distance. For the purpose of the current study, two sensors were mounted on a boom of adjustable height and attached to a tractor as shown in **Figure 1**. The sensors are mounted at a nadir angle, with scans occurring along the crop row. Measurements are collected across the crop rows as the tractor moves. Detailed information on the boom and its implementation within field plot trials is provided in Walter et al. (2019). For the current study all LiDAR measurements were collected from a single direction of travel, as opposed to the serpentine manner described in Walter et al. (2019). To investigate the repeatability of LiDAR measurements at Roseworthy, three scans were conducted at each timepoint. Two scans were collected from the same direction of travel, to observe the repeatability of duplicate scans, with the third scan collected from the opposite direction of travel, to observe any effects of travel direction on scan data.

Scans were captured at a speed of 2 km/h with the LiDAR sensor capturing data in a 70° nadir field of view, at 300 Hz, with an angular resolution of 0.133° and a theoretical scanning resolution of 1.5 mm between consecutive scans. Sensors were configured to output data to laptop computers in the tractor cab. Data capture was triggered by a 1.5 V pulse, output from a Trimble FM1000 RTK GPS unit (Trimble Inc., Sunnyvale, California, USA). This allowed individual plots to be identified *in-situ* using shapefiles created using GIS software MiniGIS (geo-konzept GmbH, Adelschlag, Germany) loaded onto the Trimble FM1000. LiDAR sensors were mounted at a height of 230 cm above the ground throughout all scans, allowing for an approximate field of view of 2 m at 80 cm above-ground level (estimated average wheat canopy height). All data collection occurred on the same day, within each sample time, with manual measurements taken immediately after plots were scanned by the LiDAR system.

### LiDAR Processing

**Figure 2:**
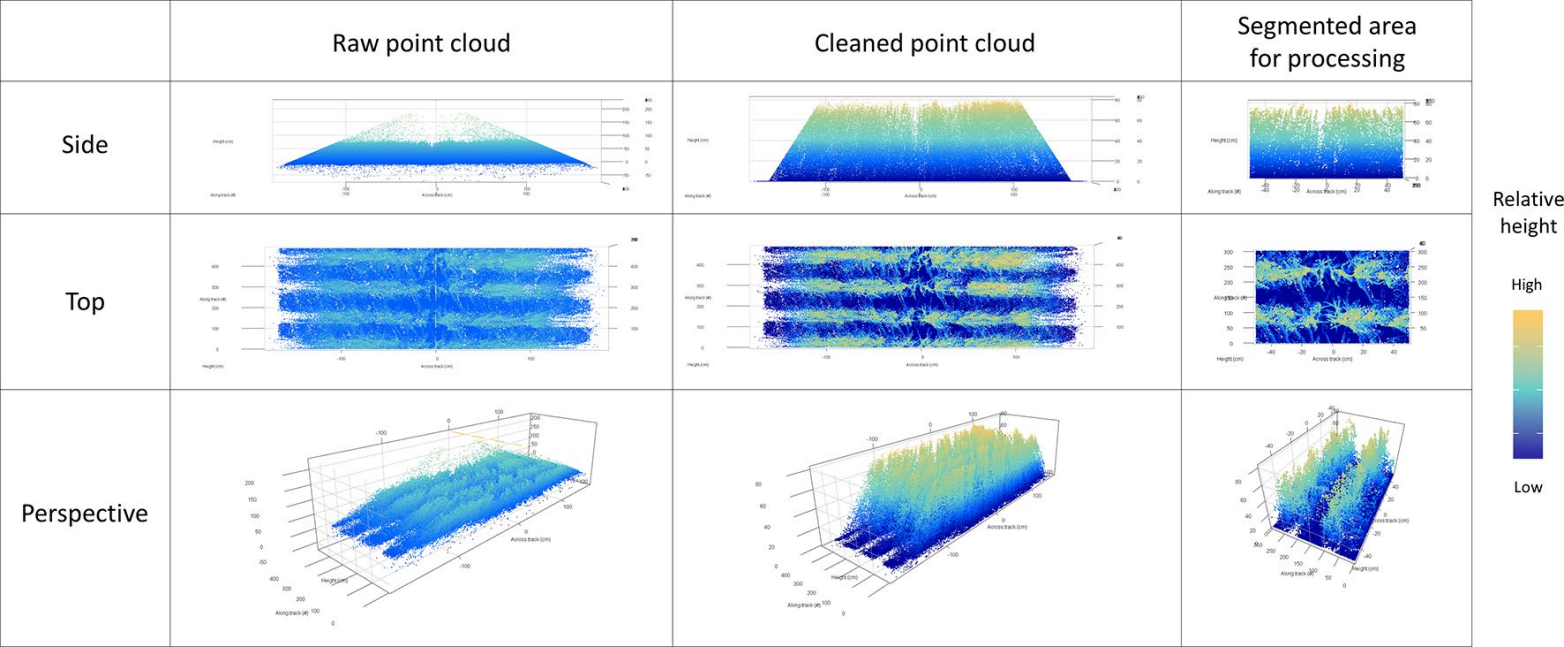
Visualisation of a point cloud collected in the current study (at Zadoks growth scale 65), showing the three steps of data processing; raw point cloud, cleaned point cloud, and segmented area used for processing of measurements, from a side, top and perspective view.

Raw scan data was processed in the R software package (R-Core Team, 2017). Scan data was cleaned to remove false returns through a process of removing negative height values and filtering each scan line through a 98^th^ percentile check to remove excessively high points. To better extract data from each plot, scanlines of two plot rows within each plot were processed, with ends of each scan trimmed to give a total plot length of 1 m (i.e. 0.5 m either side of the sensor), equating to the area of plot to be manually sampled. Points with a height less than 5 cm were re-assigned a height of 0 cm to eliminate returns from raised soil along seeding furrows, rocks or other miscellaneous objects. Visualization of these point cloud processing steps are shown in **Figure 2**. Similar procedures, for the removal of ground-level points, when dealing with fixed-height LiDAR data, have been demonstrated by Friedli et al. (2016); Sun et al. (2017) and Jimenez-Berni et al. (2018).

**Figure 3:**
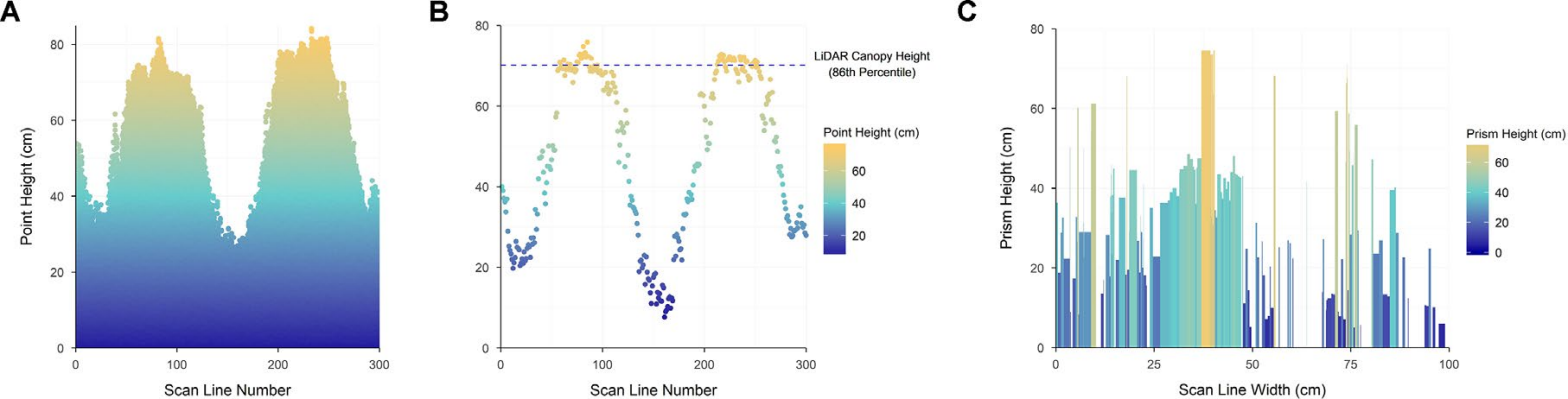
The height of all laser returns present in each scan line of a point cloud segmented for processing **(A)**, a visualization of the LiDAR Canopy Height calculation process **(B)** showing the 98^th^ percentile of processed points within each scan line and the over-all LiDAR Canopy Height value, represented with a dashed blue line, as calculated by the 86^th^ percentile of these points, and a two-dimensional representation of LiDAR Projected Volume calculated for a single scan line of the point cloud **(C)**; the width and height of each prism being represented by the *x* axis (scan line width) and the *y* axis (prism height) respectively. The depth of each prism is calculated from the distance between scan lines as the LiDAR sensor moves, producing the unplotted *z* axis (prism depth).

Canopy height was extracted through percentile algorithm in R (R-Core Team, 2017). Firstly, identifying the 98^th^ percentile of maximum returned height in each scan line (**Figure 3A**), and secondly taking the 86^th^ percentile of these values to provide an estimate of overall canopy height, henceforth referred to as LiDAR Canopy Height (LCH) (**Figure 3B**). The 86^th^ percentile was selected through optimization of Pearson’s correlation coefficient and RMSE between LCH and CH for all sample times at Roseworthy (**Supplementary Material**).

As a surrogate to AGB, plot volume estimates were produced by calculating the distance between each point in a scan line, the distance between scan lines and the height of each point. Using these three variables, a rectangular prism was created for each point in the point cloud, and volume of this prism calculated. A two-dimensional representation of these prisms for a single scan line is presented in **Figure 3C** with the *z* axis distance for these prisms provided by the movement of the LiDAR sensor. The summation of all prism volumes from within the point cloud was used as an estimate of plot volume. This measure will henceforth be referred to as LiDAR Projected Volume (LPV), as it encompasses all space below the LiDAR returns, rather than purely the area occupied by plant material. In the current study this volume is calculated as m^3^/m^2^, as this can be directly compared to plant material per square meter of plot, as measured in kg/m^2^ for AGB. A single automated script was written in R to clean raw data and simultaneously calculate LCH and LPV, with processing taking approximately 13 s per plot.

In addition to the LPV calculations, point clouds for the Roseworthy data set were processed using the formulas described by Jimenez-Berni et al. (2018) to calculate their 3D profile index (3DPI) used to estimate AGB. This index is based around the fraction of points present throughout the point cloud, rather than a volume-based measurement. It requires splitting the point cloud into layers, applying a correction factor to each layer and finally taking a summation of the corrected point fractions present in each later. This process, and the required formula, are described in detail by Jimenez-Berni et al. (2018). The processing and AGB estimation methods of Jimenez-Berni et al. (2018) were followed, using the separate equations for pre- and post-anthesis measurements presented, and finally estimating AGB through transforming LiDAR data with the linear regression equation between AGB and 3DPI at each measurement time.

### Statistical Analysis

All statistical analyses were conducted in the R software package (R-Core Team, 2017). Mixed linear models were used for multivariate analyses, comparing traits and trait collection methods, using ASREML (Gilmour et al., 2015). From multivariate analyses Pearson’s correlation coefficients were calculated between traits (raw correlations), along with genetic and residual correlations, accounting for the proportion of variance observed between the two traits based on genetic and residual components, respectively (Falconer, 1960). Outputs of multivariate analyses were also used for the calculation of broad-sense heritability (Equation 1), which can be described as the proportion of observed trait variation attributable to genetics (Visscher et al., 2008), for the traits CH, LCH, and LPV. Outputs from a randomized complete block analysis with ASREML were used to calculate broad-sense heritability for AGB.

(Equation 1.) $$H^{2}=\frac{\sigma_{\text{G}}^{2}}{\sigma_{\text{G}}^{2}+\sigma_{\text{E}}^{2}}$$

where H^2^ is broad-sense heritability, $\sigma_{\text{G}}^{2}$ is the variance attributable to genetic effects and $\sigma_{\text{E}}^{2}$ the variance attributable to environmental effects (residual variance).

Due to the small sample size and large spatial spread of AGB measurements collected within trials at each sample time, genetic and residual correlations were not calculated between AGB and other traits.

## Results

### LiDAR Repeatability

**Figure 4:**
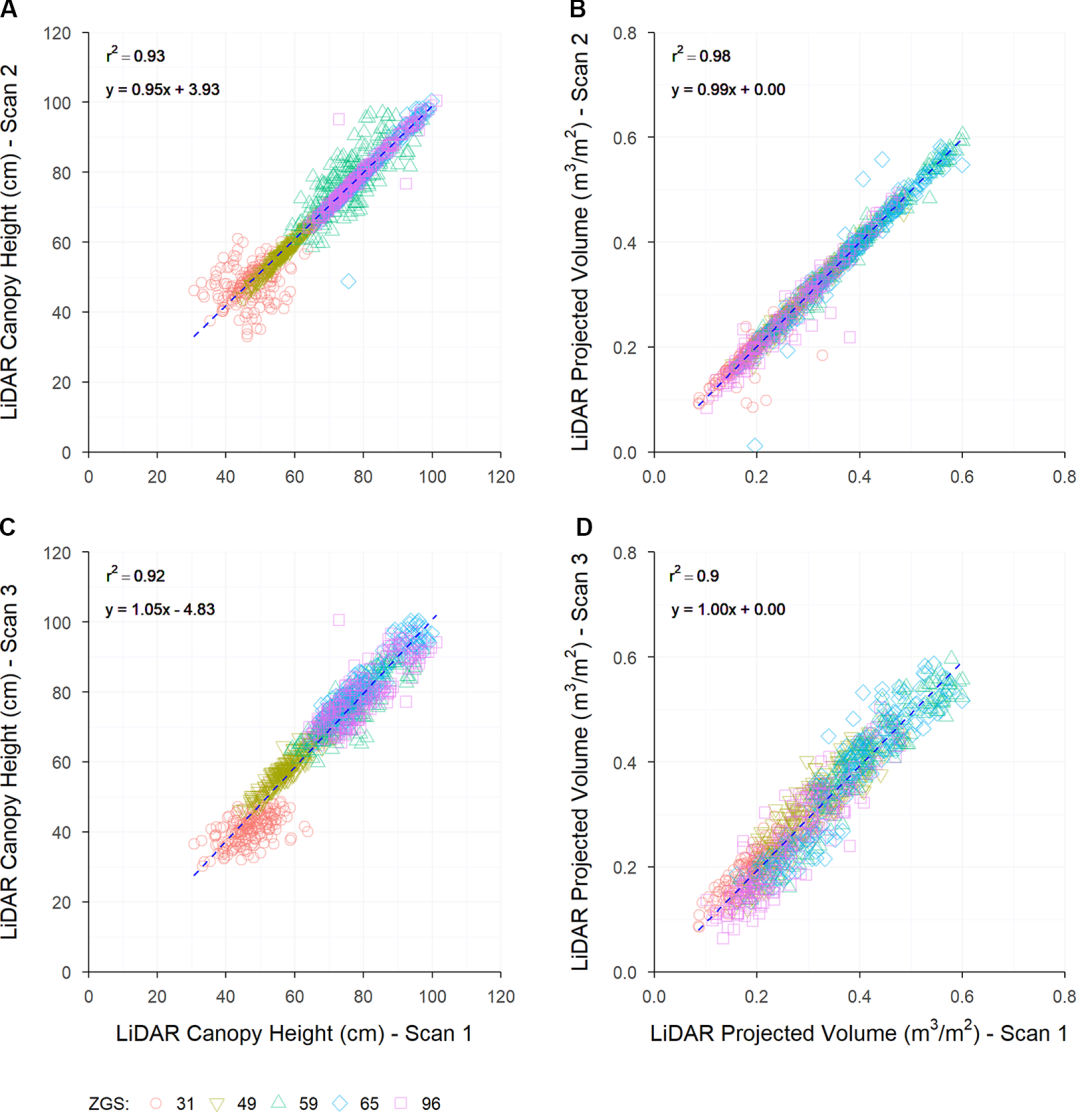
The repeatability of LiDAR-based measurements collected at Roseworthy throughout the season, comparing scans collected from the same direction **(A**, **B)** and the opposite direction **(C**, **D)** for the measurement of LiDAR canopy height **(A**, **C)** and LiDAR projected volume **(B**, **D)**. Dashed lines indicate the line of best fit, coloured shapes indicate measurements collected at Zadoks growth scales (ZGS) as follows, red circles at ZGS 31, yellow triangles at ZGS 49, green triangles at ZGS 59, blue diamonds at ZGS 65 and pink squares at ZGS 90.

Repeatability of multiple measurements taken at Roseworthy was generally high for both LCH and LPV measurements at both individual sample times (**Table 3**) and when pooling sample times (**Figure 4**). Scans taken in the same direction of travel show greater repeatability than scans taken in opposite directions of travel.

**Table 3:** Coefficient of determination (r^2^) and components (slope ± standard error, and intercept ± standard error) for linear regression models between repeated scans in the same and opposite directions, processed for the traits LiDAR Canopy Height (LCH) and LiDAR Projected Volume (LPV), at each sample time (ZGS) at Roseworthy.

Trait	Scan 1 vs Scan 2 (Same direction)				Scan 1 vs Scan 3 (Opposite Direction)			
	ZGS	r^2^	Slope ± s.e.	Intercept ± s.e.	ZGS	r^2^	Slope ± s.e.	Intercept ± s.e.
**LCH**	31	0.08	0.31 ± 0.08	31.98 ± 3.67	31	0.31	0.71 ± 0.08	17.73 ± 3.21
	49	0.99	0.97 ± 0.01	1.32 ± 0.46	49	0.88	0.97 ± 0.03	1.76 ± 1.50
	59	0.61	0.72 ± 0.04	20.02 ± 3.28	59	0.76	0.93 ± 0.04	5.58 ± 2.84
	65	0.95	0.94 ± 0.02	5.15 ± 1.28	65	0.86	0.95 ± 0.03	2.45 ± 2.26
	96	0.95	0.98 ± 0.02	1.38 ± 1.36	96	0.68	0.87 ± 0.04	11.14 ± 3.47
**LPV**	31	0.82	0.92 ± 0.03	0.01 ± 0.00	31	0.75	0.93 ± 0.01	0.01 ± 0.01
	49	0.99	0.99 ± 0.01	0.00 ± 0.00	49	0.79	0.82 ± 0.04	0.04 ± 0.01
	59	0.99	0.99 ± 0.01	0.00 ± 0.00	59	0.91	0.88 ± 0.04	0.04 ± 0.01
	65	0.96	0.94 ± 0.01	0.02 ± 0.00	65	0.89	0.82 ± 0.05	0.05 ± 0.01
	96	0.95	0.95 ± 0.02	0.01 ± 0.00	96	0.84	0.81 ± 0.05	0.05 ± 0.00

All models are significant at the level of p < 0.001.

Repeatability of height measurements was seen to be extremely high at three of the five sample times, ZGS 49, 65, and 96, when scanned in the same direction of travel, with high r^2^ values, linear regression coefficients nearing one and intercepts nearing zero (**Table 3**). In contrast repeatability of samples taken at ZGS 31 and 59 showed much greater variation, with lower linear regression coefficients and intercepts further away from zero (**Table 3**, **Figure 4A**). Repeatability of measurements in opposite directions of travel was less accurate than the same direction of travel (**Figure 4C**). Generally, measurements of LCH from opposite directions had linear regression coefficients nearing one and intercepts nearing zero, though lower r^2^ values than scans from the same direction (**Table 3**), with differences between timepoints being less pronounced. A similar trend for repeatability of LPV measurements was also observed, though overall LPV showed greater reproducibility than LCH. High repeatability was observed between LPV measurements in the same direction of travel (**Figure 4B**) and good repeatability in opposite directions of travel (**Figure 4D**).

### Canopy Height

**Figure 5:**
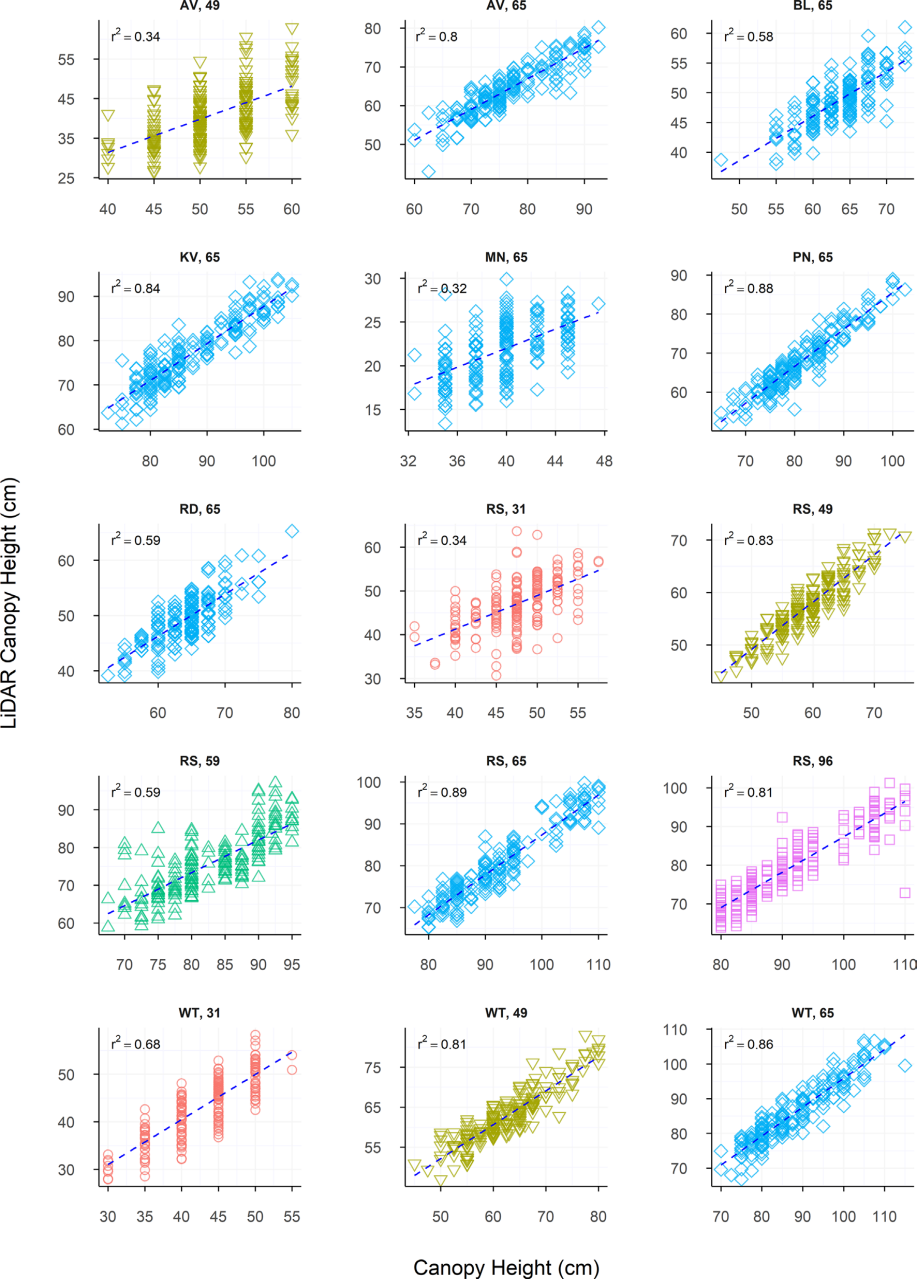
The relationship between LiDAR Canopy Height and Canopy Height, presented individually for each site and sample time (ZGS). Dashed lines indicate the line of best fit, coloured shapes indicate measurements collected at Zadoks growth scales (ZGS) as follows, red circles at ZGS 31, yellow triangles at ZGS 49, green triangles at ZGS 59, blue diamonds at ZGS 65 and pink squares at ZGS 90.

**Figure 6:**
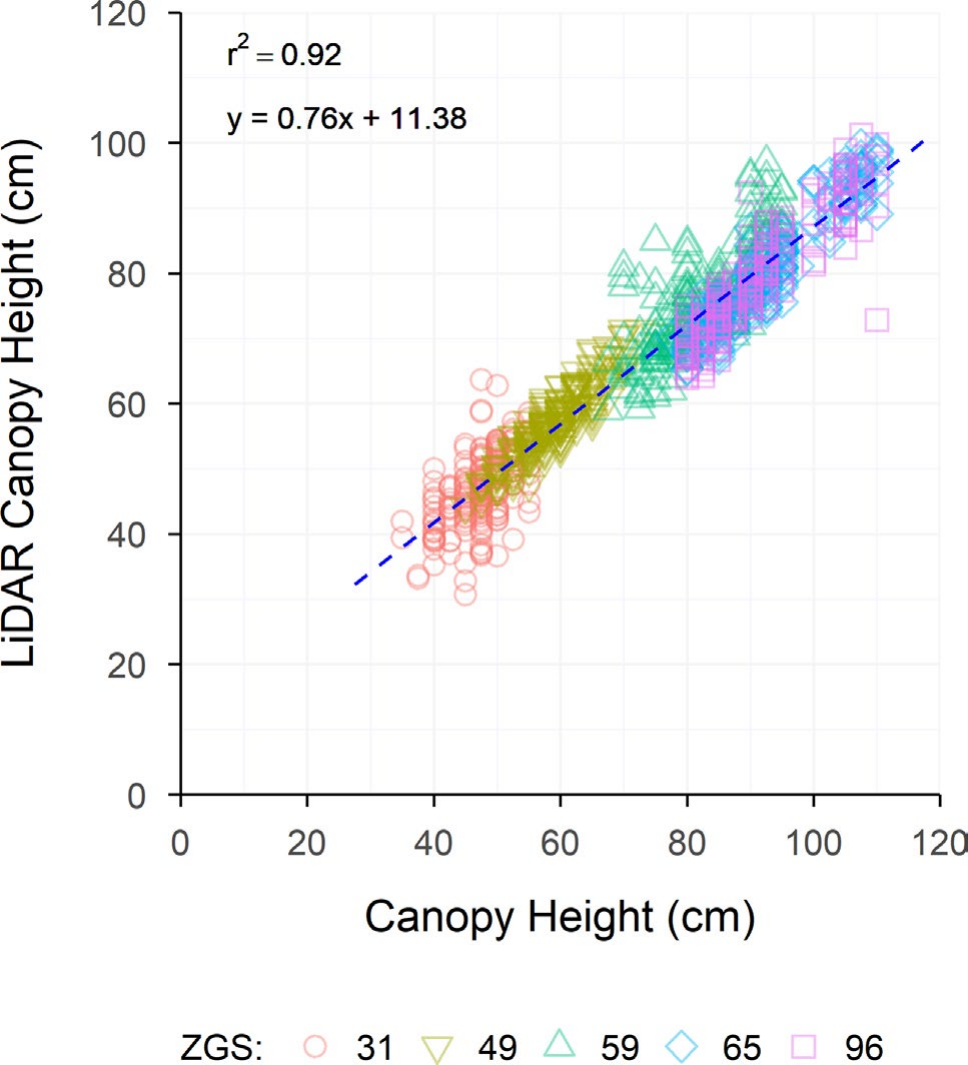
The relationship between LiDAR Canopy Height and Canopy Height for all sample times measured at Roseworthy. Dashed lines indicate the line of best fit, coloured shapes indicate measurements collected at Zadoks growth scales (ZGS) as follows, red circles at ZGS 31, yellow triangles at ZGS 49, green triangles at ZGS 59, blue diamonds at ZGS 65 and pink squares at ZGS 90.

A wide range in canopy height was observed between site locations and sample timepoints, with this being especially apparent between environments. Strong raw correlations were observed between LCH and manually measured height for all sites (r = 0.56–0.94), and at the majority of sample times (**Table 4**). However, individual sample times at some sites showed poorer correlation compared to the rest of the data set. Strong linear relationships were observed between CH and LCH at the majority of sites (**Figure 5**), though weaker relationships were observed for some early growth stages (Roseworthy at ZGS 31 and Angas Valley at ZGS 49), or when CH was low (Minnipa ZGS 65). Pooling measurements throughout the season showed strong continuity of data and strong linear relationships between CH and LCH, such as presented in **Figure 6** for Roseworthy.

**Table 4:** Correlations between traits, plus or minus standard error, at each site and sample time (ZGS), measured in the current study.

Site	ZGS	Correlation											
		Raw			Raw		Genetic		Residual		Raw	Genetic	Residual
		AGB	AGB	AGB	LPV	LPV	LPV	LPV	LPV	LPV	CH	CH	CH
		*LPV*	*CH*	*LCH*	*CH*	*LCH*	*CH*	*LCH*	*CH*	*LCH*	*LCH*	*LCH*	*LCH*
**AV**	49	0.86 ± 0.11 ***	0.64 ± 0.16 ***	0.68 ± 0.16 ***	0.52 ± 0.06 ***	0.87 ± 0.04 ***	0.68 ± 0.24 ***	0.92 ± 0.08 ***	0.48 ± 0.06 ***	0.87 ± 0.02 ***	0.58 ± 0.06 ***	0.88 ± 0.10 ***	0.48 ± 0.06 ***
	65	0.64 ± 0.16 ***	0.16 ± 0.21	0.28 ± 0.20	0.67 ± 0.06 ***	0.76 ± 0.05 ***	0.89 ± 0.09 ***	0.92 ± 0.06 ***	0.44 ± 0.07 ***	0.73 ± 0.04 ***	0.90 ± 0.03 ***	0.98 ± 0.01 ***	0.67 ± 0.04 ***
**BL**	65	0.69 ± 0.16 ***	-0.15 ± 0.21	0.21 ± 0.21	0.30 ± 0.07 ***	0.62 ± 0.06 ***	0.84 ± 0.13 ***	0.81 ± 0.14 ***	0.17 ± 0.07 ***	0.63 ± 0.05 ***	0.76 ± 0.05 ***	1.00 ± 0.01 ***	0.42 ± 0.06 ***
**KV**	65	0.45 ± 0.19 *	0.25 ± 0.20	0.22 ± 0.21	0.67 ± 0.05 ***	0.80 ± 0.04 ***	0.88 ± 0.09 ***	0.89 ± 0.08 ***	0.33 ± 0.07 ***	0.67 ± 0.04 ***	0.91 ± 0.03 ***	1.00 ± < 0.01 ***	0.51 ± 0.06 ***
**MN**	65	0.70 ± 0.15 ***	0.71 ± 0.15 ***	0.69 ± 0.15 ***	0.29 ± 0.07 ***	0.80 ± 0.04 ***	0.64 ± 0.25 ***	0.88 ± 0.10 ***	0.04 ± 0.08	0.74 ± 0.03 ***	0.56 ± 0.06 ***	0.91 ± 0.08 ***	0.08 ± 0.08
**PN**	65	0.66 ± 0.16 ***	0.39 ± 0.20	0.42 ± 0.20 *	0.77 ± 0.05 ***	0.86 ± 0.04 ***	0.90 ± 0.07 ***	0.92 ± 0.06 ***	0.55 ± 0.05 ***	0.88 ± 0.02 ***	0.94 ± 0.03 ***	1.00 ± < 0.01 ***	0.58 ± 0.05 ***
**RD**	65	0.66 ± 0.16 ***	0.58 ± 0.17 **	0.72 ± 0.15 ***	0.58 ± 0.06 ***	0.82 ± 0.04 ***	0.74 ± 0.18 ***	0.80 ± 0.14 ***	0.26 ± 0.07 ***	0.72 ± 0.04 ***	0.77 ± 0.05 ***	0.99 ± 0.01 ***	0.45 ± 0.06 ***
**RS**	31	0.86 ± 0.11 ***	0.58 ± 0.17 **	0.71 ± 0.15 ***	0.70 ± 0.05 ***	0.83 ± 0.04 ***	0.45 ± 0.34 ***	0.62 ± 0.26 ***	0.53 ± 0.06 ***	0.86 ± 0.02 ***	0.83 ± 0.04 ***	0.97 ± 0.04 ***	0.62 ± 0.05 ***
	49	0.73 ± 0.15 ***	0.70 ± 0.15 ***	0.70 ± 0.15 ***	0.76 ± 0.05 ***	0.90 ± 0.03 ***	0.93 ± 0.06 ***	0.97 ± 0.03 ***	0.50 ± 0.06 ***	0.87 ± 0.02 ***	0.90 ± 0.03 ***	1.00 ± < 0.01 ***	0.64 ± 0.05 ***
	59	−0.05 ± 0.21	0.21 ± 0.21	0.04 ± 0.21	0.60 ± 0.07 ***	0.72 ± 0.06 ***	0.64 ± 0.23 ***	0.83 ± 0.12 ***	0.20 ± 0.08 ***	0.64 ± 0.05 ***	0.77 ± 0.05 ***	0.95 ± 0.04 ***	0.28 ± 0.07 ***
	65	0.62 ± 0.17 **	0.35 ± 0.2	0.51 ± 0.18 *	0.65 ± 0.07 ***	0.73 ± 0.06 ***	0.82 ± 0.13 ***	0.77 ± 0.16 ***	0.25 ± 0.09 ***	0.71 ± 0.05 ***	0.94 ± 0.02 ***	1.00 ± < 0.01 ***	0.29 ± 0.07 ***
	96	0.47 ± 0.19 *	−0.01 ± 0.21	0.06 ± 0.21	0.22 ± 0.10 *	0.31 ± 0.10 **	0.18 ± 0.37 *	0.19 ± 0.37 **	0.25 ± 0.11 ***	0.28 ± 0.11 ***	0.90 ± 0.03 ***	1.00 ± < 0.01 ***	0.27 ± 0.07 ***
	31	0.75 ± 0.14 ***	0.44 ± 0.19 *	0.27 ± 0.21	0.63 ± 0.06 ***	0.56 ± 0.06 ***	0.82 ± 0.13 ***	0.84 ± 0.11 ***	0.31 ± 0.07 ***	0.70 ± 0.04 ***	0.58 ± 0.06 ***	0.96 ± 0.03 ***	0.24 ± 0.07 ***
**WT**	49	0.55 ± 0.18 **	0.70 ± 0.15 ***	0.66 ± 0.16 ***	0.74 ± 0.05 ***	0.86 ± 0.04 ***	0.84 ± 0.11 ***	0.91 ± 0.07 ***	0.29 ± 0.08 ***	0.81 ± 0.03 ***	0.91 ± 0.03 ***	0.99 ± 0.01 ***	0.41 ± 0.07 ***
	65	0.47 ± 0.19 *	0.22 ± 0.21	0.37 ± 0.20	0.65 ± 0.06 ***	0.74 ± 0.06 ***	0.82 ± 0.13 ***	0.81 ± 0.13 ***	0.44 ± 0.07 ***	0.71± 0.04 ***	0.93 ± 0.03 ***	1.00 ± < 0.01 ***	0.56 ± 0.05 ***

Raw correlations shown for Above-ground Biomass (AGB), LiDAR Projected Volume (LPV), Canopy Height (CH) and LiDAR Canopy Height (LCH); raw, genetic and residual correlations between LiDAR Projected Volume, Canopy Height and LiDAR Canopy Height; and raw genetic and residual correlations between Canopy Height and LiDAR Canopy Height. Significance of each correlation is indicated as *p < 0.05, **p <0.01 and ***p < 0.001.

Raw (r = 0.56 to 0.94) and genetic (r_g_ = 0.91 to 1.00) correlations between CH and LCH were strong across all sample times, while residual correlations ranged from 0.08 to 0.67 (**Table 4**). For repeated measures there were no apparent trends for raw, genetic or residual correlations over time. Both CH and LCH had high heritability at all times of measurement, excluding Winulta at ZGS 31. Heritability tended to increase over time at sites where repeated measurements were taken (**Table 5**).

**Table 5:** Broad-sense heritability of each trait, at each site and sample time (ZGS), measured in the current study.

Trait	Site and sample time														
	AV		BL	KV	MN	PN	RD	RS					WT		
	*49*	*65*	*65*	*65*	*65*	*65*	*65*	*31*	*49*	*59*	*65*	*96*	*31*	*49*	*65*
Above-ground biomass	0.47	0.22	0.46	0.32	0.67	0.26	0.48	0.52	0.12	0.42	0.78	0.59	0.63	0.33	0.45
LiDAR projected volume	0.32	0.79	0.48	0.76	0.61	0.78	0.75	0.58	0.80	0.89	0.90	0.83	0.33	0.57	0.76
Canopy height	0.76	0.90	0.76	0.97	0.82	0.94	0.76	0.89	0.91	0.97	0.98	0.98	0.49	0.86	0.95
LiDAR canopy height	0.67	0.86	0.78	0.93	0.84	0.95	0.82	0.87	0.95	0.97	0.98	0.96	0.41	0.84	0.94

### Above-Ground Biomass

**Figure 7:**
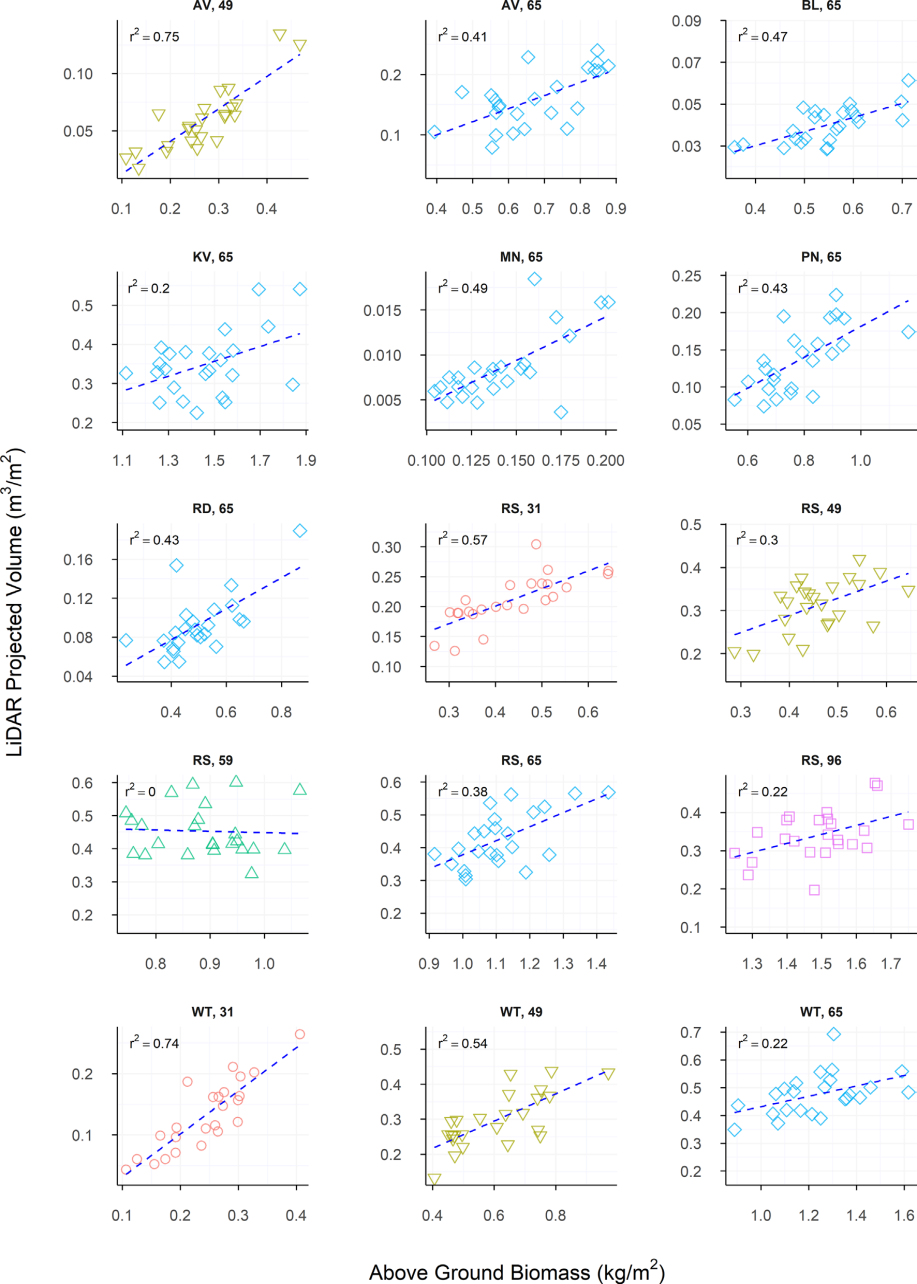
The relationship between LiDAR Projected Volume and Above-ground Biomass, presented individually for each site and sample time measured in the current study. Dashed lines indicate the line of best fit, coloured shapes indicate measurements collected at Zadoks growth scales (ZGS) as follows, red circles at ZGS 31, yellow triangles at ZGS 49, green triangles at ZGS 59, blue diamonds at ZGS 65 and pink squares at ZGS 90.

**Figure 8:**
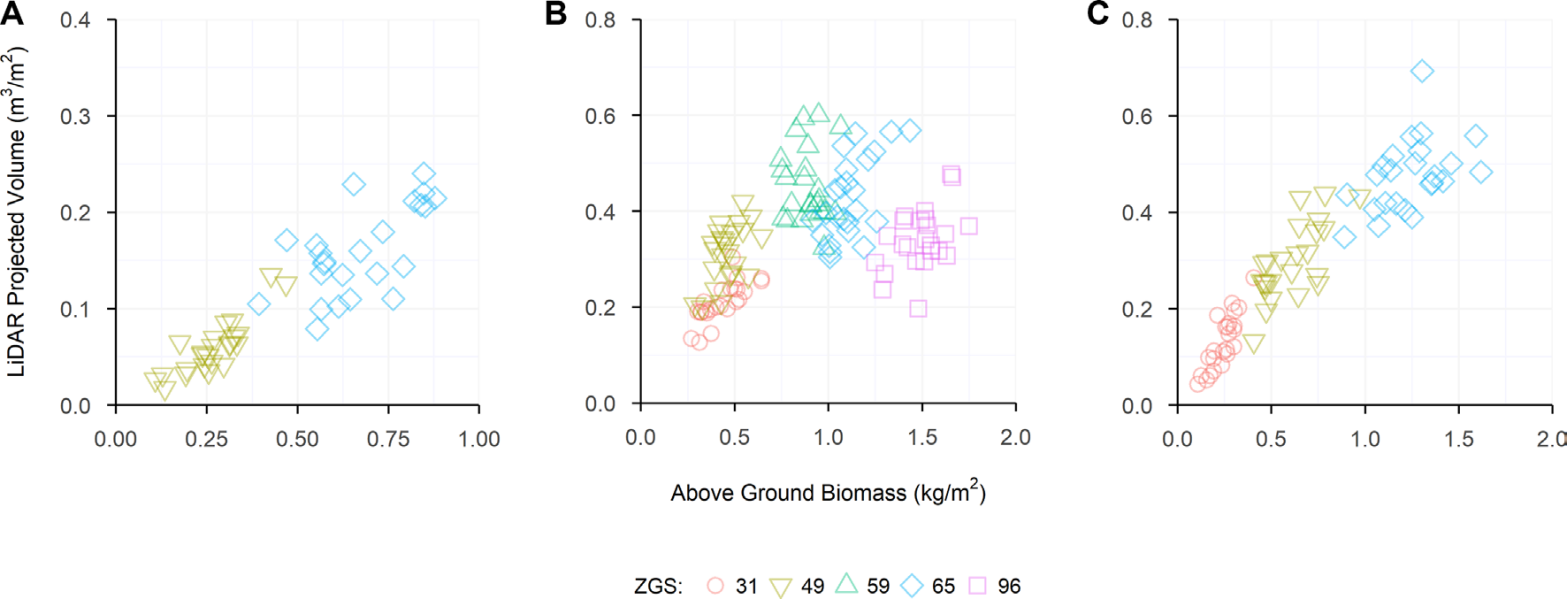
The relationship between LiDAR Projected Volume and Above-ground Biomass for each sample time collected at Angas Valley **(A)**, Roseworthy **(B)** and Winulta **(C)**. Coloured shapes indicate measurements collected at Zadoks growth scales (ZGS) as follows, red circles at ZGS 31, yellow triangles at ZGS 49, green triangles at ZGS 59, blue diamonds at ZGS 65 and pink squares at ZGS 90.

Above-ground biomass samples collected showed large amounts of variation between sites, with LPV showing similar amounts of variation. Raw correlations between AGB and LPV were predominantly strong and positive, though some weaker correlations were observed, with one weak negative correlation (**Table 4**). **Figure 7** shows the linear nature of the relationship between AGB and LPV within each sample. The relationships between measurements of AGB and LPV collected over the growing season at Angas Valley, Roseworthy and Winulta are displayed in **Figure 8**. Both Angas Valley and Winulta showed an increase of AGB and LPV over time. This was also observed at Roseworthy for most sample times. However, samples collected at ZGS 65 and 96 showed increased AGB (compared to previous samples) but did not show any increase in LPV, with slight decreases in LPV being observed.

Above-ground biomass correlated most strongly to LPV for much of the raw data, though a number sites showed stronger, or similar, correlations to CH and LCH (**Table 4**). LiDAR projected volume correlated strongly to LCH for most measurements. Similar but generally weaker correlations were observed between LPV and CH.

The heritability of AGB measurements was generally lower than that of LPV measurements, although this was reversed in some instances. Heritability of AGB appears to show no trend across repeated measures, though heritability of LPV appears to generally increase over time, with the exception of Roseworthy at ZGS 96.

**Figure 9:**
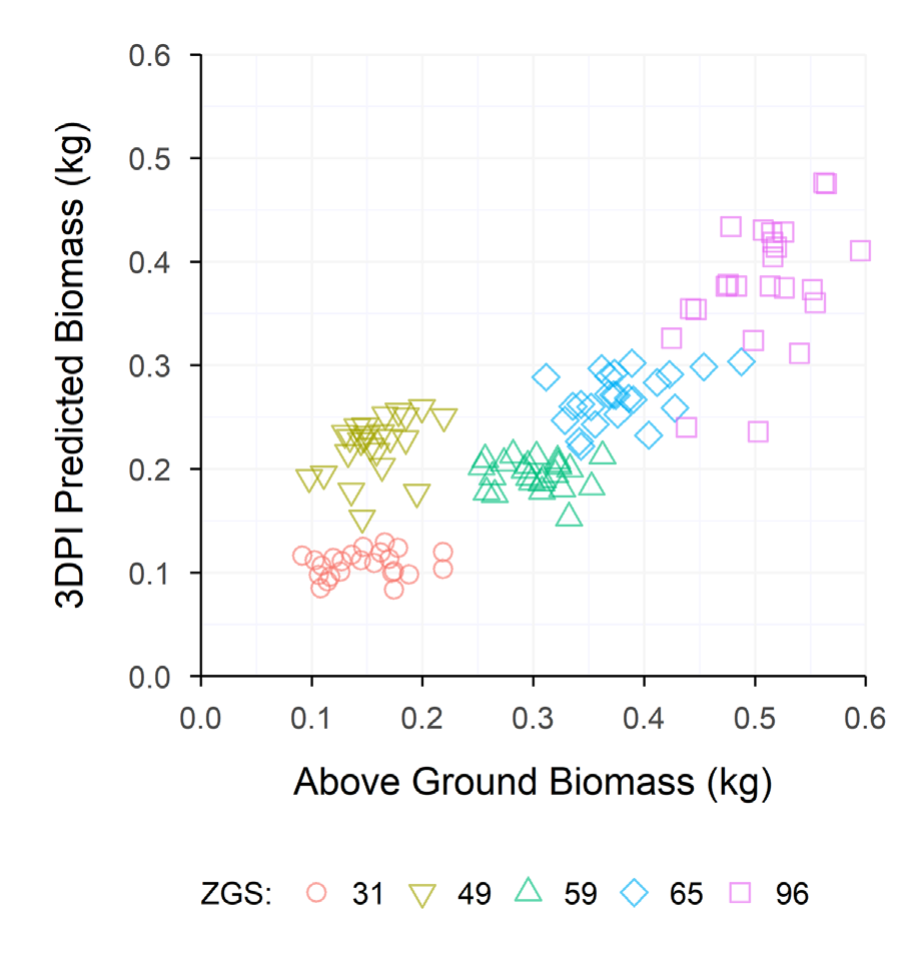
The relationship between 3DPI Biomass and manually measured Above-ground Biomass, for each sample time at Roseworthy. Coloured shapes indicate measurements collected at Zadoks growth scales (ZGS) as follows, red circles at ZGS 31, yellow triangles at ZGS 49, green triangles at ZGS 59, blue diamonds at ZGS 65 and pink squares at ZGS 90.

To assess the effectiveness of the LPV measurements calculated in the current study as an AGB estimator, LPV was compared to 3DPI, as described by Jimenez-Berni et al. (2018), for the Roseworthy data set. Except for ZGS 31, LPV was strongly correlated with 3DPI and, in general, showed slightly greater correlations to AGB (**Table 6**). A strong relationship between AGB and 3DPI-predicted AGB was observed throughout the season (**Figure 9**), excluding the ZGS 49 measurement which did not fit this trend. A similar relationship was observed by Jimenez-Berni et al. (2018).

**Table 6:** Pearson’s correlation coefficients (r), between LiDAR Projected Volume (LPV), 3DPI and Above-ground Biomass (AGB), for each sample time (ZGS) at Roseworthy.

ZGS	LPV: AGB		3DPI: AGB		LPV: 3DPI	
31	0.75 ± 0.14	***	0.17 ± 0.21		0.05 ± 0.07	
49	0.55 ± 0.18	**	0.42 ± 0.19	*	0.82 ± 0.04	***
59	−0.05 ± 0.21		−0.07 ± 0.75		0.88 ± 0.03	***
65	0.62 ± 0.17	**	0.44 ± 0.19	*	0.90 ± 0.03	***
96	0.47 ± 0.19	*	0.48 ± 0.19	*	0.95 ± 0.02	***

Values are shown as ± standard error. Significance of each correlation is indicated as *p < 0.05, **p <0.01 and ***p < 0.001.

## Discussion

The adoption of LiDAR and terrestrial laser scanners (TLS) as field-based sensors for the non-destructive phenotyping of AGB and canopy height has been discussed and demonstrated numerous times in the literature (Deery et al., 2014; Tilly et al., 2014; Eitel et al., 2016; Friedli et al., 2016; Kronenberg et al., 2017; Sun et al., 2017; Virlet et al., 2017; Jimenez-Berni et al., 2018; Sun et al., 2018), ultimately contributing towards a solution to the phenotyping bottleneck present in large-scale research and plant breeding programs (Cobb et al., 2013; Araus and Cairns, 2014). Despite the different approaches to the deployment of these sensors, there are still many questions left unanswered, particularly with regard to the robustness and reliability of the data collected and its application and value within research or field crop breeding programs.

In the current study, adaption of the imaging boom described in Walter et al. (2019) to accommodate a dual LiDAR system allowed for LiDAR sensors to be efficiently deployed across eight large-scale wheat breeding trial sites in a range of environments, and for large amounts of point cloud data to be collected at multiple growth stages.

### Data Repeatability

Objective and repeatable data collection is of key importance within breeding and research programs but can be difficult to obtain through traditional in-field measurements. Thus, the overall high repeatability and objective nature of point cloud data collected with LiDAR sensors in the current study shows great potential for integration within field-based research programs.

Repeated LiDAR scans from the same direction of travel were capable of producing near identical LCH measurements for three of the five physiological growth stages measured (ZGS 49, 65, and 96). The remaining two growth stages (ZGS 31 and 59) still showed sound repeatability of LCH measurements, though not to the extent seen at other growth stages. Interestingly, the repeatability of LPV across all physiological growth stages showed a strong relationship, with few outlying points, similar to that observed for LCH at ZGS 49, 65, and 96. There is no apparent cause of the variation observed between LCH measurements at ZGS 31 and 59 and we can only speculate that this is an artefact of the LCH calculation process, as the variation between LPV measurements for these samples is much lower than for LCH and is similar to that observed for other LPV measurements.

Measurements of both LCH and LPV were less repeatable when measured with opposite directions of travel, however repeatability was still strong. The discrepancies observed between measurements collected in opposite directions likely arise for two reasons; firstly, despite endeavors to mount LiDAR sensors identically on each side of the boom, there are likely to be small differences between the two, altering the laser emission and return pattern of each unit. Secondly, triggering of the LiDAR sensors relative to shapefiles on the RTK GPS unit requires calibration, which if not precise may result in small variations to the area of plot measured. Towards addressing these issues, LiDAR sensor and direction of travel could be fitted as random terms within spatial analyses, which may help to account for variation between measurements.

To the authors’ best knowledge, repeatability of data derived from LiDAR plot scans has yet to be described in the literature, however Busemeyer et al. (2013) have reported very high repeatability of canopy height measurements collected with a light-curtain (r^2^ = 0.99, Mean Relative Error = 0.01). Given the similarities between the type of data obtained from these two sensors, the high repeatability of LiDAR data observed in the current study is a positive, but not unexpected, result.

### Canopy Height

Point cloud data collected through the LiDAR system was able to accurately and repeatedly estimate canopy height of wheat grown in field plots across multiple growth stages and environments. Strong raw correlations were observed at the majority of locations and growth stages measured, ranging from r = 0.56 at the weakest, to r = 0.94 at the strongest. Similar results have been previously reported for wheat and other field crops, with r^2^-values of 0.99 (Jimenez-Berni et al., 2018), 0.99 (Kronenberg et al., 2017), 0.99 (Virlet et al., 2017), 0.97 (Sun et al., 2018), 0.91 (Tilly et al., 2014), 0.87 (Eitel et al., 2016), 0.84 (Walter et al., 2018) and 0.73 to 0.93 (Friedli et al., 2016). The current study and those of Jimenez-Berni et al. (2018) and Sun et al. (2018) collected data with mobile LiDAR systems deployed in field; Virlet et al. (2017) used a dual 3D laser scanner system mounted on the Field Scanalyzer gantry; Kronenberg et al. (2017) used a cable suspended laser scanner mounted on the ETH field phenotyping platform; Tilly et al. (2014); Eitel et al. (2016) and Friedli et al. (2016) used TLS systems; and Walter et al. (2018) used digital cameras and photogrammetry. Though each of these systems differ, comparable results have been achieved from each, reinforcing the concept that 3D data collected in the form of point clouds is highly suitable for the derivation of canopy height.

While the correlations presented in the current study do not appear to be as strong as some previously reported in the literature, it is important to consider the way in which the data has been collated for presentation. Data from the current study has been collected from eight wheat varieties across eight locations and in some circumstances at multiple time points. This contrasts with the data presented by Jimenez-Berni et al. (2018), where three replicates of 18 genotypes were measured at a single location and time point, with the mean values of each genotype being presented and used for correlation. The results presented by Jimenez-Berni et al. (2018) are more similar to those from **Table 4**, of genetic correlations between CH and LCH at each site, which showed improved correlations compared to raw data.

The weakest correlation between CH and LCH in the current study occurred at Minnipa, where severe drought conditions occurred during most of the 2017 growing season. Very little variation was observed between canopy heights, with varieties ranging from 32.5 to 47.5 cm, much less than the variation observed at other sites. A similar explanation is likely for the weaker correlations present at Roseworthy at ZGS 31 and Angas Valley at ZGS 49, where plants measured early in the growing season had short canopies and little variation in CH. These correlations, as well as all correlations in the current study, could likely be improved through optimisation of the percentile algorithm used to process the data. A similar process has been described by Friedli et al. (2016), with data in **Supplementary Material** reinforcing this work, and showing the variability in selecting an algorithm based on maximizing the correlation and reducing the RMSE. The authors believe the use of a single algorithm is suitable in large-scale breeding or research programs as it is generally not feasible to collect ground truth data for each site and timepoint to optimize this process. Moreover, the implications of taking physical measurements are counterintuitive to the aims of deploying these sensor systems for rapid collection of large amounts of data.

The similarity in the heritabilities calculated for CH and LCH gives great confidence in LiDAR-derived canopy height, showing that in terms of accuracy/repeatability within a breeding program it is as good as, or in some cases superior to, manual measurements. In addition to the high heritability of LCH demonstrated in the current study, a similarly high heritability of LiDAR-derived CH has previously been reported by Kronenberg et al. (2017) for a diverse set of European bread wheat cultivars (H^2^ = 0.96), though this was not compared to the heritability of manual measurements. While in the current study, and in that of Kronenberg et al. (2017), heritabilities were calculated for material containing greater variation in CH than often present within breeding populations, it is expected these results are still directly applicable as CH is known to be a highly heritable trait. The strong genetic correlations observed between CH and LCH in the current study further support that LCH will be suitable for estimating CH within breeding populations, as similar genetic components are measured by both methods. The moderately strong residual correlations between CH and LCH would seem to indicate the ability of LCH to capture differences in CH resulting from environmental variance across the experimental area. This makes sense from a physiological perspective, as plant height can be influenced by a number of biotic and abiotic factors, which may result in uneven growth throughout the trial. While the variation observed in CH within the current study was typically greater than would be observed within modern breeding populations, the results of current study suggest that LiDAR sensors would be suitable for measuring relative CH, or for measuring absolute CH if required. The slopes of lines of best fit for **Figures 5** and **6** show that as CH increased LCH underestimated CH, generally by around 10 cm. This is likely due to the data cleaning process and LCH algorithm function, and though it is not an issue if relative CH is desired, if an absolute measurement of CH is required this discrepancy will need to be accounted for.

### Above-Ground Biomass

The LPV measurement in the current study has been shown capable of estimating a wide range of AGB, across different varieties, phenological stages and environments. To date, very few studies have investigated the use of point cloud data for the type of bio-volume measurements presented here. The few that have presented data for different plant species, such as cotton (Sun et al., 2018), arctic shrubs (Greaves et al., 2015) and trees (Rosell Polo et al., 2009), or for wheat which was grown in a single environment (Jimenez-Berni et al., 2018; Walter et al., 2018), except for one study by Eitel et al. (2014), where two adjacent fields of wheat plots with differing micro-climates were investigated. The collection of the point cloud data across eight different environments in the current study is an important addition to the current understanding of AGB estimation from point cloud bio-volume measurements.

The multi-location measurements presented in the current study provide a unique set of results, where large ranges in AGB were observed across a single phenological growth stage. At each location there was a moderately strong correlation between AGB and LPV, with each of these relationships (excluding Roseworthy at ZGS 59) being suitably explained by a linear regression model (**Figure 7**).

Combining repeated measurements from within sites at Angas Valley, Roseworthy and Winulta showed heteroscedastic relationships, which appear to be curvilinear. This is most apparent at Roseworthy, where AGB increases with time, however LPV plateaus and declines following ear emergence (ZGS 59). This trend also appears to be occurring at Angas Valley and Winulta, where AGB seems to be increasing more than LPV, though the final sample occurring at ZGS 65 for these sites prevents confirmation of this. This can likely be explained by the senescence of the crop. As the crop senesces, leafy volume is lost through leaves drying out and contracting, however, overall AGB continues to increase due to grain fill. Though this curvilinear relationship can be explained, it does highlight the limitation of using volume as an estimator for AGB at later growth stages. A further limitation of using LPV for AGB estimation may be present in dense canopies, where laser penetration within the canopy is poor. In such cases an over-estimation of volume will occur, as only points collected from the top of the canopy will be used to compute LPV. While this did not appear to be a limitation in the current study, with the laser sufficiently penetrating the canopy at maximum leafy biomass, it should be noted as a potential limitation of LiDAR-based volume measurements in high AGB environments. Furthermore, different relationships are observed over time at Angas Valley, Roseworthy and Winulta, suggesting that unique curvilinear relationships may be required for each environment. Considering geographical differences as a predictor of seasonal differences, it is likely these relationships will also alter from year to year as a result of the differing abiotic and biotic factors which occur between seasons and environments. This in turn may affect numerous aspects of crop morphology, which may alter the relationship between volume-based measurements and AGB. This introduces the question of how breeders will use such data. Will it be used to measure AGB over time within trials, comparing relative AGB at a single time point between trials, or for comparing relative AGB at individual time points? To better understand the interactions occurring between volume-based measurements and AGB, and how these interactions may influence a breeder’s use of these measurements, a series of multi-year, multi-environment trials would be beneficial.

The moderately strong correlations observed between AGB and LPV within individual measurement points, align with the results of Walter et al. (2018) and Jimenez-Berni et al. (2018), where linear regressions provided a suitable explanation for the relationship between AGB and point cloud bio-volume estimates. The work of Jimenez-Berni et al. (2018) is the most comparable to the current study, and their results indicate a strong linear relationship between AGB of wheat and their 3D Indices of processed LiDAR data for numerous physiological growth stages. However, optimization of equations was conducted for the processing of these 3D Indices, based on developmental stage, which were further transformed using separate equations for pre and post-anthesis measurements for comparison to measured AGB. These processes of optimisation require ground-truth data, which as discussed previously, are not likely to be collected within a breeding program. It is also worth noting that in the current study, and that of Jimenez-Berni et al. (2018), as AGB increases, digital measurements obtained with LiDAR sensors correlate less strongly to manual measurements, with the pooling of measurements showing a heteroscedastic relationship. This is likely a limitation imposed by the LiDAR sensors used in these studies, which return only a single discrete point, compared with units capable of returning multiple discrete points or a full wave form. Capturing multiple discrete returns or the full wave form, may overcome this issue and allow for deeper penetration within the crop canopy. However, such systems are currently prohibitively expensive for their deployment within plant breeding programs, both from an upfront cost and from a data processing perspective.

Processing LiDAR data from Roseworthy using 3DPI as described by Jimenez-Berni et al. (2018), yielded a positive linear relationship across all sample times (**Figure 9**). The results presented here align with those of Jimenez-Berni et al. (2018), and show the robustness of their 3DPI when applied to an alternate data set, even in the absence of optimisation. The sample at ZGS 49 did fall outside of the linear relationship observed for 3DPI, however, similar results were observed for ZGS 49 in the processing methods of the current study, where samples at ZGS 49 did not increase in projected volume but did increase in AGB. Values of 3DPI at individual sample times correlated strongly with LPV, with the exception of the ZGS 31 sample, which showed a weak correlation (**Table 6**). 3DPI also showed similar, but slightly weaker, correlations to AGB compared to LPV (**Table 6**). It is likely these correlations could be improved through the optimization of the *k* value within the 3DPI equation. However, as described for canopy height, continued optimization of such equations runs contrary to the benefits of implementing such phenotyping systems within field crop breeding programs. For this reason, we believe the performance of the LPV measurement used within the current study is applicable to field crop breeding programs, providing sound estimates of AGB and requiring no optimization for deployment within breeding programs. Similar volume based measurements to those used in the current study were successfully utilized to estimate cotton AGB by Sun et al. (2018), who observed manually-measured biomass to correlate strongly to volume measurements across a small number of plots.

Above-ground biomass of cereal crops is highly variable (Sharma, 1993); which was confirmed in the current study where H^2^ ranged from 0.12 to 0.78. Despite this broad range, only at two measurement times (Roseworthy at ZGS 31 and Angas Valley at ZGS 49) did AGB have a substantially greater heritability than LPV, while for all other measurements LPV showed similar, or substantially greater heritability than AGB. This generally high heritability of LPV, combined with the moderate correlations to AGB, indicates that it could be used as an effective tool for making genetic gain if selecting for AGB.

### Application of Data

Past studies investigating the use of LiDAR sensors as a phenotyping tool have shown strong correlations to manually-collected data for multiple traits and have suggested potential applications of such data. Despite these often strong relationships, practical applications have yet to be published. For this point cloud generated phenotype data to be used effectively within wheat breeding programs, data collection and processing needs to be quick and largely automated, reducing the manual labor and time required. While the travel speed of the LiDAR system was relatively slow in the current study (2 km/h) this can be easily increased with alterations to the system hardware, specifically the path from GPS signal to LiDAR sensor trigger. Despite this, throughput of the system allows two unique plots to be scanned every 3 s, allowing for 2,400 plots to be scanned per hour. To the author’s knowledge the throughput of similar ground-based LiDAR systems has not previously been reported in the literature, with the exception of Sun et al. (2018) who reported an approximate throughput of 600 plots per hour for cotton field plots of similar size to the plots measured in the current study. While the current reported throughput of 2,400 plots per hour is high, there is still potential to improve upon this by increasing the travel speed of the system in conjunction with alterations to the system hardware. It is expected this could increase throughput to approximately 7,400 plots per hour, as reported for the HIB described by Walter et al. (2019). However, it should be noted that were travel speed to be increased, longitudinal resolution of collected point clouds would decrease. As such, further validation for correlations between LiDAR-based and manual measurements would be required at greater speeds of travel. Processing of the data takes approximately 13 s per plot (though this could potentially be optimized for greater speed) resulting in a total time of approximately 15 s to collect and convert raw data into LCH and LPV measurements for a single plot. This is an immense increase in throughput compared to manual methods, with CH measurements taking approximately 10 s per plot and AGB cuts several minutes per plot, not including time required for handling, drying and weighing samples post collection. Further to this, all measurements taken with the system are non-destructive, allowing for repeated measurements in season and for AGB to be estimated without impacting upon plot grain yield. This now provides the opportunity for breeders to collect large-scale data sets for AGB, which were previously impossible to collect due to the destructive nature of manual measurements.

Even though in the current study, the LiDAR system was effectively able to provide large increases in throughput and decreases in manual labor for the collection of CH and AGB measurements, it is likely that within a large-scale breeding program, collection of this data would only occur at one or two key physiological time points throughout the season. Examples of this could be; once the greater part of a site has reached first node (ZGS 31) for estimating early AGB, or at anthesis (ZGS 65) for estimating maximum AGB, though these timepoints would be driven by the specific trait of interest. For routine integration within a wheat breeding program, these measurements would ideally be combined with another field operation, such as herbicide or fungicide spraying. Combining data collection with routine field maintenance practices would allow for repeated measurements during the season, while also reducing the logistical burden of transporting equipment between field sites. Alternatively, a more focused set of measurements could be conducted at a single site, allowing many repeated measures throughout the season. However, this would fail to assess the genotype-by-environment interaction effects which need to be considered by breeders. Ultimately the field campaign undertaken will depend on the breeding objectives of the program and consequently the specific data desired by the breeder. The data presented on LiDAR data repeatability in the current study suggests that LiDAR sensors could be used to measure absolute values of CH and to a lesser extent AGB. However, it seems the most apparent fit for such data within breeding programs is for the relative measurement of these traits, which could be used to select within populations to achieve the desired breeding objective.

The LiDAR-based data generated in the current study shows great promise for application within breeding programs, particularly as the heritability of LCH and LPV assessments were generally comparable to, or greater than, manual measurements, indicating that genetic gain can be made through selection of each trait. There are many examples as to how the type of data collected within the current study could be applied within wheat breeding programs: one example is the selection of breeding lines based on early AGB accumulation at first node (ZGS 31). Some programs may wish to select for this trait, or against it, depending on the desired purpose of the material, e.g. for dual-purpose wheats (i.e. those producing large amounts of grazeable biomass prior to ZGS 31) or for weed competitiveness. A second example is for breeders wishing to select for increased AGB independently of CH. Currently to achieve this, breeders must manually collect measurements of CH and AGB. However, this process can be greatly simplified as LCH and LPV are calculated from the same data, and a combination of the two measurements could be used for the selection of increased AGB while maintaining lower CH. Broadening the scope of potential application, there are many other field crop breeding programs which could take advantage of the type of data presented in the current study, prime examples being biomass heavy crops, such as those used for hay or silage, as well as horticultural breeding programs where leafy biomass or volume may be key traits.

## Conclusion

Through the deployment of a mobile ground-based LiDAR system across multiple environments within a large-scale commercial wheat breeding program, it has been shown that the collection and processing of 3D point cloud data is highly repeatable, strongly correlated to manual measurements of CH and AGB, and highly heritable. This combination makes LiDAR sensors a promising and valuable tool for wheat or other field crop breeders who wish to non-destructively measure CH and or AGB within their breeding programs.

Discussion on the application of LiDAR sensors to breeding programs in the current study has been based around the direct or indirect selection of specific traits within breeding programs, however there are also the exciting possibilities of fitting LiDAR data in multivariate analyses of yield trials, or within crop physiological models, in both cases to improve upon current techniques of data analysis and variety performance prediction. The authors suggest that the possibilities listed above are the logical progression for future work investigating LiDAR sensors, either for use in breeding or research programs.

## Data Availability

The datasets generated for this study are available on request to the corresponding author.

## Author Contributions

JW, JE and HK: designed and oversaw the management of experiments; JW: established data processing methods, collected and analyzed all data; JW, JE, GM and HK: interpreted analyzed data; JW: wrote the manuscript with contributions from JE, GM and HK.

## Funding

The authors wish to acknowledge the South Australian Grains Industry Trust (UA514), the Grains Research and Development Corporation (GRS11009), The University of Adelaide School of Agriculture, Food and Wine, and the Australian Government Research Training Program Scholarship for their funding support of this research.

## Conflict of Interest Statement

JW, JE, and HK are affiliated with Australian Grain Technologies Pty Ltd, a commercial plant breeding company. The remaining author declares that the research was conducted in the absence of any commercial or financial relationships that could be construed as a potential conflict of interest.
