# Supplementary material for: Estimating Biomass and Canopy Height With LiDAR for Field Crop Breeding
Source: Front Plant Sci. 2019 Sep 26;10:1145. doi: 10.3389/fpls.2019.01145 (PMC6775483; doi:10.3389/fpls.2019.01145)
Supplement: Supplementary file 1 [file DataSheet_1.pdf]

![frontiers]

# *Supplementary Material*

## Algorithm Selection for LiDAR Canopy Height

Algorithms ranging from the 60th to 99th percentile height across scan lines were compared across all sample times and repeated measures at the Roseworthy site. Each sample time contains 192 observations. Values form each of these algorithms were correlated to their corresponding manual measurement, with the correlation coefficients and the root mean square error (RMSE) of the data, from all time points, being compared in Supplementary Figures 1 and 3 respectively. Due to the reversed relationship between percentile algorithm, correlation coefficients and RMSE for measurements during the first sample time (ZGS 31), these measurements were not included in determining the mean correlation coefficient or RMSE across the data sets. Supplementary Table 1 below indicates which plots relate to which sample time and measurement.

**Supplementary Table 1.** Labels of scans presented in Supplementary Figures 1 and 3, with the associated Zadoks Growth Scale (ZGS) and scan direction.

| ZGS | Scan 1 (Forward) | Scan 2 (Forward) | Scan 3 (Reverse) |
|---|---|---|---|
| *31* | t11 | t12 | t13 |
| *49* | t21 | t22 | t23 |
| *59* | t31 | t32 | t33 |
| *65* | t41 | t42 | t44 |
| *96* | t51 | t52 | t55 |

The 89th percentile algorithm produced the greatest mean correlation coefficient, while the 86th percentile algorithm produced the minimum mean RMSE. Looking at Supplementary Figures 2 and 4 it is apparent there is no large peak for either mean statistic, and there is likely very little difference in selecting percentile algorithms within a 5-percentile range. With this is mind the 86th percentile algorithm was selected to broadly represent crop canopy height, and henceforth data referred to as LiDAR Canopy Height (LCH) in the current study was produced with this algorithm.

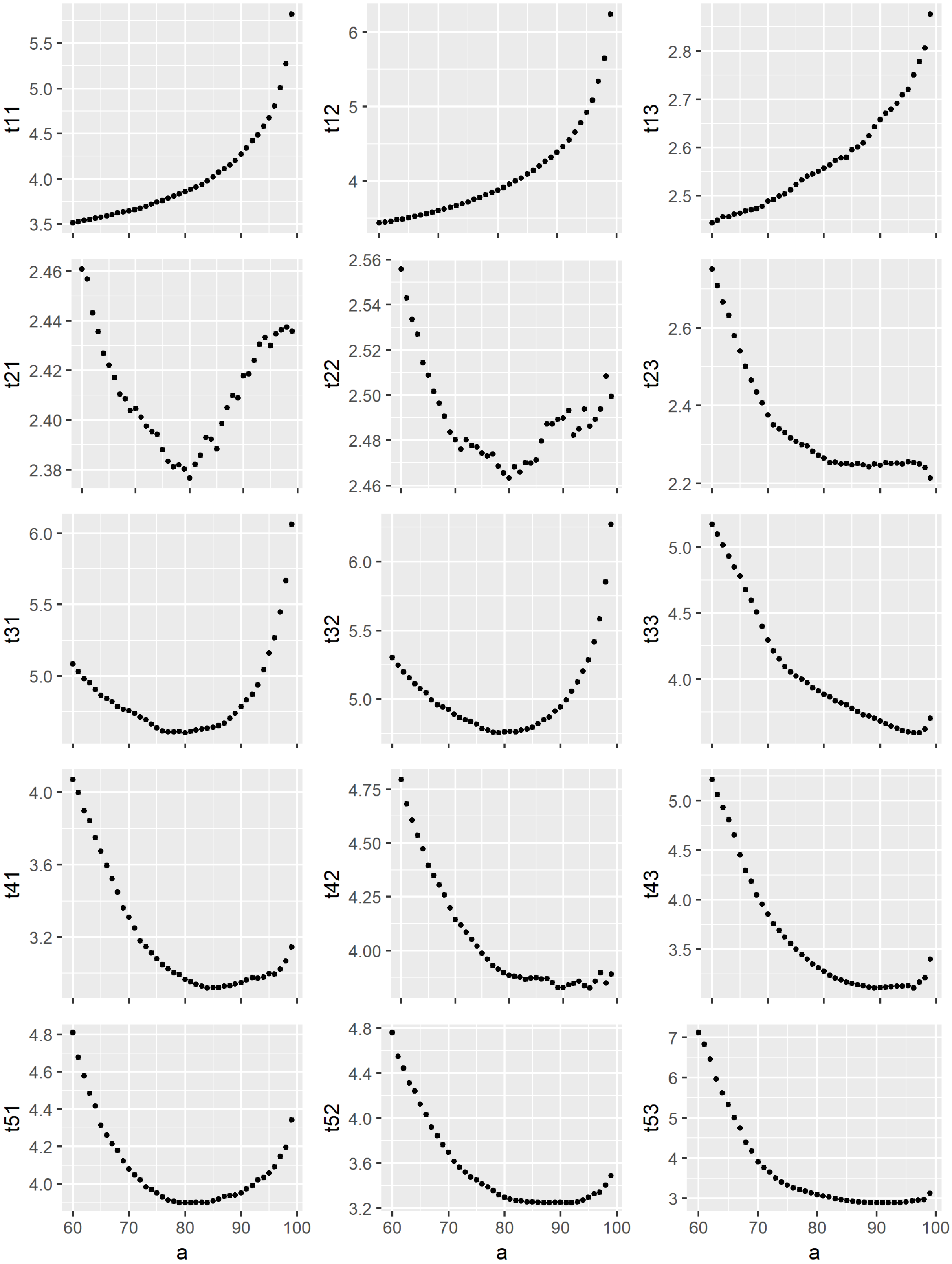


**Supplementary Figure 1.** RMSE between manually measured canopy height and LiDAR measured canopy height, for percentile algorithms ranging from the 60th to 99th percentile. Where figures are labelled as sample time and LiDAR sample repetition (1: forward, 2: forward, 3: reverse). RMSE is presented on the Y axis, with percentile algorithm on the X axis.

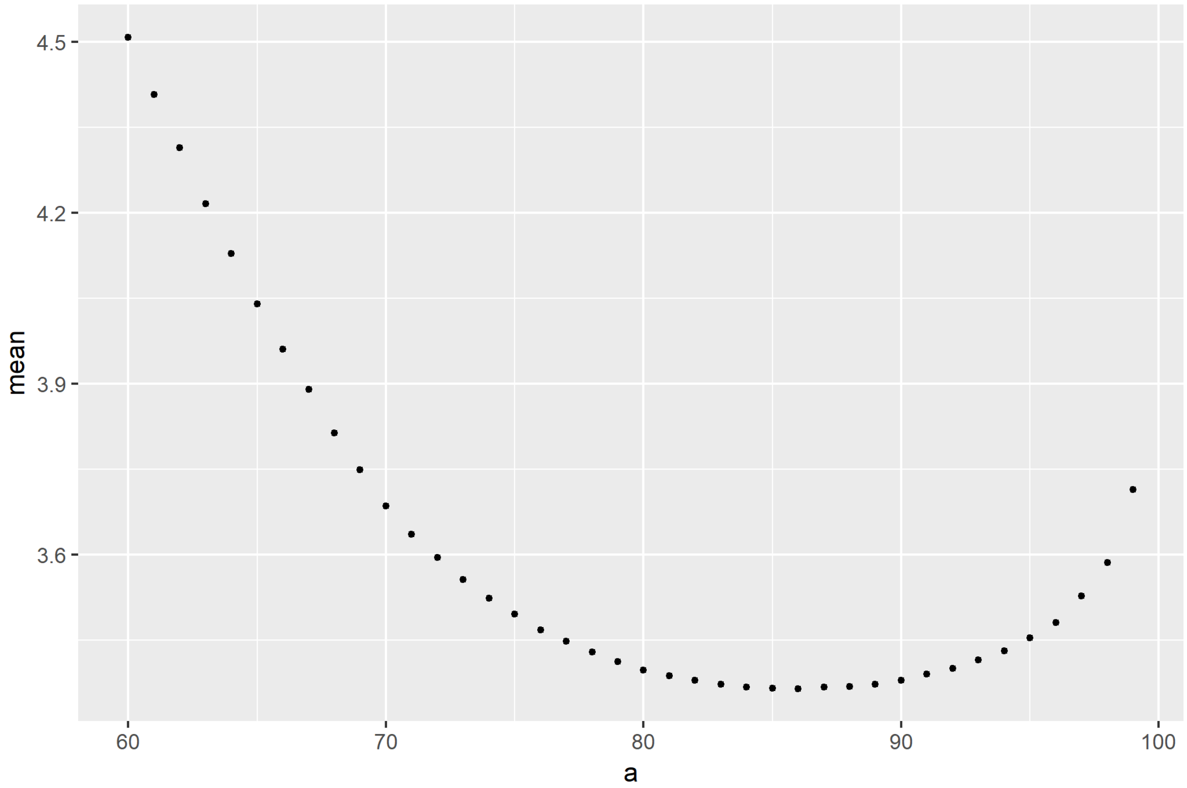


**Supplementary Figure 2.** Mean RMSE across all sample times, excluding t11, t12 and t13, of manually measured canopy height and LiDAR measured canopy height, for percentile algorithms ranging from the 60$^{th}$ to 99$^{th}$ percentile. RMSE is presented on the Y axis, with percentile algorithm on the X axis.

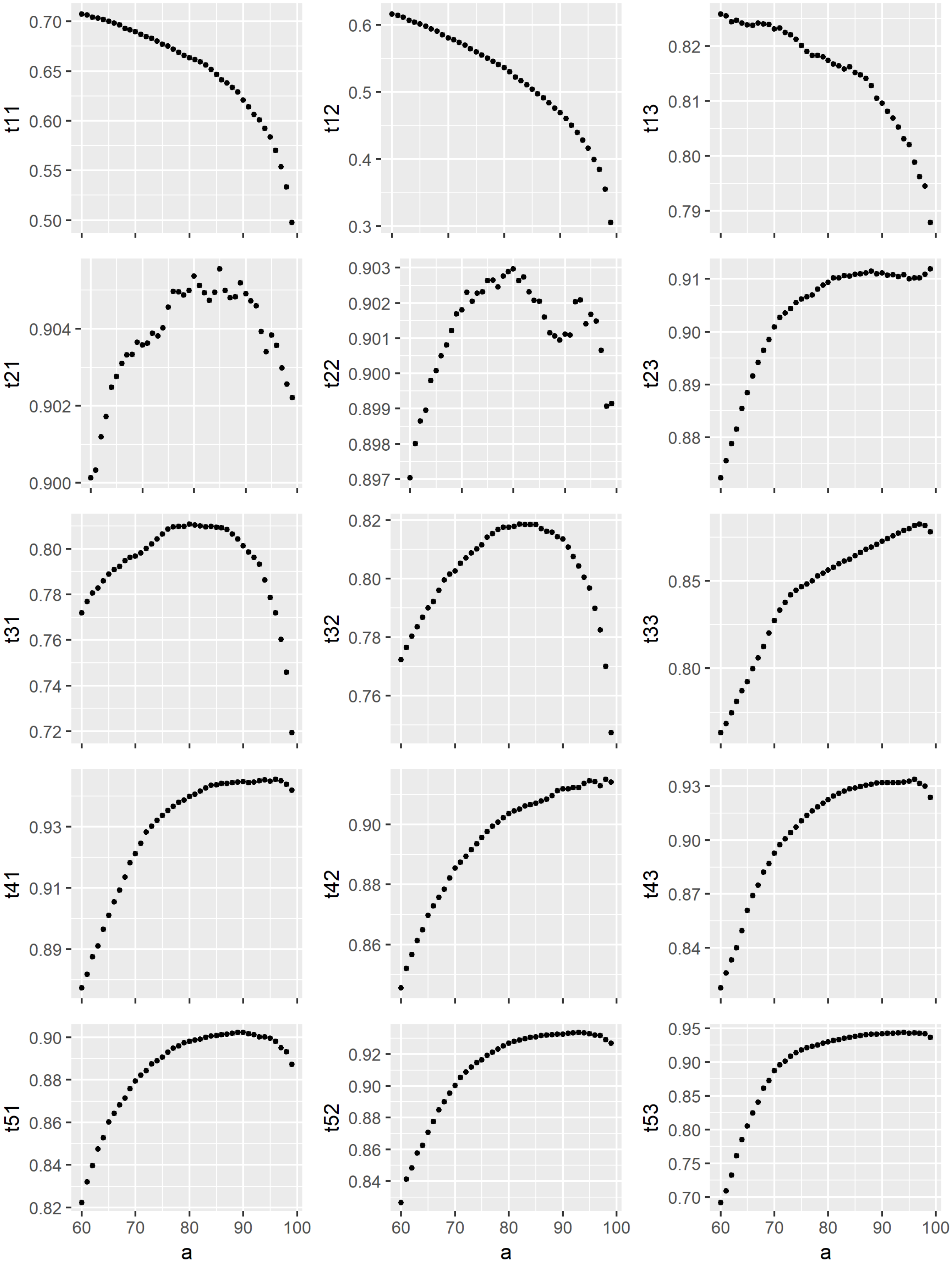


**Supplementary Figure 3.** Pearson's correlation coefficients between manually measured canopy height and LiDAR measured canopy height, for percentile algorithms ranging from the 60th to 99th percentile. Where figures are labelled as sample time and LiDAR sample repetition (1: forward, 2: forward, 3: reverse). Correlation coefficient is presented on the Y axis, with percentile algorithm on the X axis.

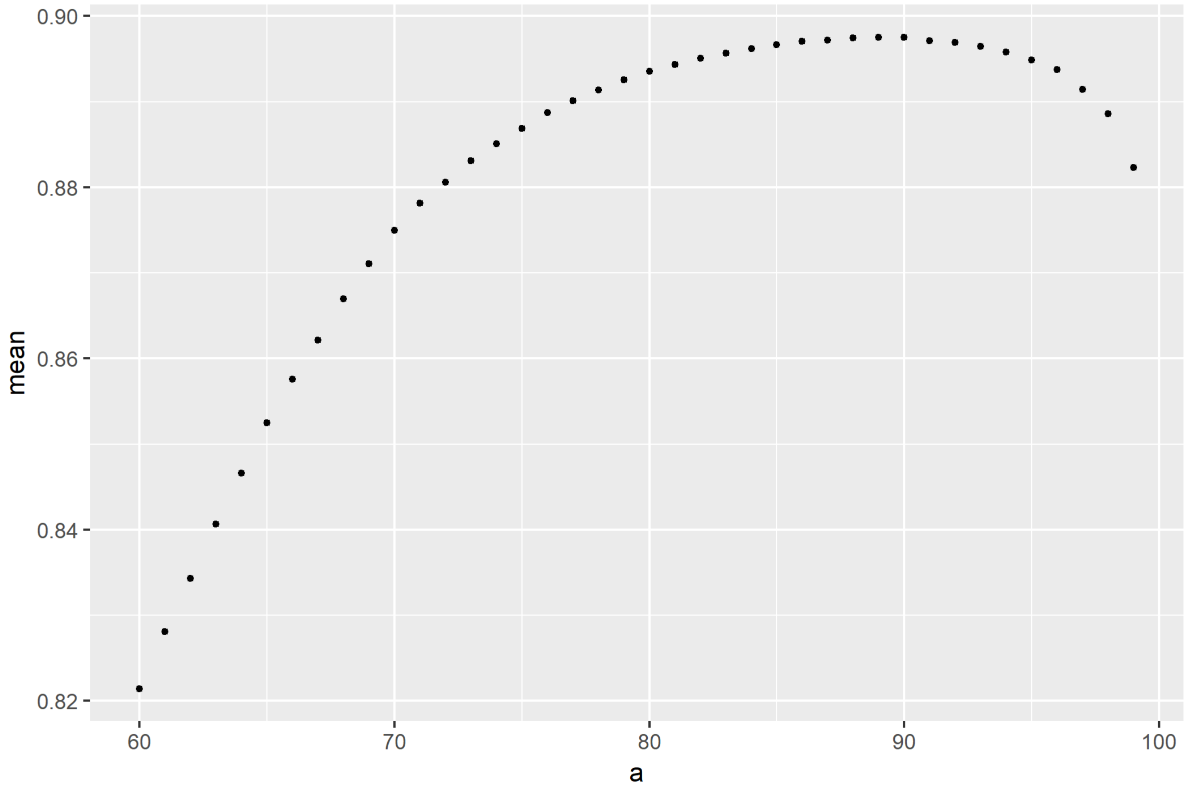


**Supplementary Figure 4.** Mean Pearson's correlation coefficient across all sample times, excluding t11, t12 and t13, of manually measured canopy height and LiDAR measured canopy height, for percentile algorithms ranging from the 60$^{th}$ to 99$^{th}$ percentile. Correlation coefficient is presented on the Y axis, with percentile algorithm on the X axis.
